# Hypertensive Disorders of Pregnancy

**DOI:** 10.14797/mdcvj.1305

**Published:** 2024-03-14

**Authors:** Courtney Newman, Victoria Petruzzi, Pedro T. Ramirez, Christopher Hobday

**Affiliations:** 1 Obstetrics and Gynecology Department, Houston Methodist, Houston, Texas, US; 2 Obstetrics and Gynecology Department, Gynecological Oncology Department, Houston Methodist, Houston, Texas, US

**Keywords:** chronic hypertension, preeclampsia, pregnancy

## Abstract

According to the American College of Obstetricians and Gynecologists (ACOG), women who have a systolic blood pressure ≥ 140 mm Hg and/or a diastolic pressure ≥ 90 mm Hg before pregnancy or before 20 weeks of gestation have chronic hypertension. Up to 1.5% of women in their childbearing years have a diagnosis of chronic hypertension, and 16% of pregnant women develop hypertension during their pregnancy. Physiological cardiovascular changes from pregnancy may mask or exacerbate hypertensive diseases during gestation, which is why prepregnancy counseling is emphasized for all patients to optimize comorbidities and establish a patient’s baseline blood pressure. This review provides an overview of the diagnoses and treatments of hypertensive diseases that can occur in pregnancy, including definitions of key terms and types of hypertension as well as ACOG recommendations.

## Introduction

Up to 1.5% of pregnant women have a diagnosis of chronic hypertension.^1,2^ Additionally, 16% of patients develop hypertension in pregnancy.^1,3^ Physiological cardiovascular changes from pregnancy may serve to mask or exacerbate hypertensive diseases in that period. Prepregnancy counseling is emphasized for all patients to optimize comorbidities and establish a patient’s baseline blood pressure. Similarly, serial laboratory exams and antenatal surveillance are needed to monitor both the mother and fetus for signs of worsening disease. Labetalol and nifedipine are safe oral antihypertensives to use throughout pregnancy to manage blood pressures. The goal blood pressure value is below 140/90 mm Hg for those with chronic hypertension. Low-dose aspirin is recommended for high-risk patients in order to help reduce the risk of preeclampsia in pregnancy. Timing of delivery depends on how well the patient’s blood pressure is controlled along with whether they meet criteria for preeclampsia, with or without severe features. Finally, the risk of cardiovascular disease and hypertension in the postpartum period in patients with hypertensive diagnoses of pregnancy should be a routine part of postpartum counseling for patients.

## Chronic Hypertension in Pregnancy

### Background and Definitions

The rate of chronic hypertension in pregnancy increased approximately 67% between 2000 and 2009, with Black women disproportionately experiencing the biggest increase (87%).^2,4^ Traditionally, chronic hypertension in pregnancy has been diagnosed when the patient has two separate blood pressure readings ≥ 140 mm Hg systolic or ≥ 90 mm Hg diastolic before pregnancy or before 20 weeks gestation. However, recent recommendations from the American College of Cardiology (ACC) and American Heart Association (AHA) define stage I chronic hypertension as systolic blood pressures between 130 and 139 mm Hg or diastolic blood pressures between 80 and 89 mm Hg, thereby increasing the number of patients with this diagnosis.^5^

Systemic vascular resistance decreases up to 30% in the first trimester of pregnancy, which typically leads to an accompanying decrease in blood pressures of up to 10%, and a typical nadir in maternal blood pressure occurs between 16 and18 weeks’ gestation.^3^ These physiologic changes in pregnancy can mask underlying chronic hypertension if there are no records of a pregnant patient’s blood pressures prior to pregnancy. In addition, these patients may be asymptomatic and unaware of their chronic disease until pregnancy or until a worsened disease state occurs. Because blood pressures return to prepregnancy levels in the third trimester, and patients with chronic hypertension can have a baseline level of proteinuria, these patients can erroneously be diagnosed with gestational hypertension and/or preeclampsia. Therefore, an appropriate clinical work-up must be performed with patients at risk.^1^

Between 20% and 50% of patients with chronic hypertension will also develop superimposed preeclampsia in pregnancy.^1,6^ Superimposed preeclampsia is defined as preeclampsia in a pregnant patient with a diagnosis of hypertension before pregnancy or before 20 weeks of gestation; this may include a sudden increase in blood pressure, newly developed proteinuria, or a change in baseline proteinuria. The risk of superimposed preeclampsia increases with the following risk factors: end-organ damage, secondary hypertension, Black race, smoking, obesity, long-standing disease (a diagnosis of hypertension for ≥ 4 years), diastolic blood pressures of ≥ 100 mm Hg, or a prior history of preeclampsia.^7^ Superimposed preeclampsia can be challenging to diagnose in pregnancy, but it typically will present as laboratory changes, including but not limited to thrombocytopenia, elevated liver function tests, and/or elevated uric acid levels. It can be difficult to distinguish a chronic hypertension exacerbation from superimposed preeclampsia because both can be associated with worsening levels of hypertension and proteinuria.^1^

Pregnant patients with chronic hypertension have up to a five- to six-times greater risk of stroke, pulmonary edema, renal insufficiency and failure, myocardial infarction, preeclampsia, placental abruption, postpartum hemorrhage, and gestational diabetes.^8^ Their fetuses and neonates have a greater risk of stillbirth or perinatal death, growth restriction, preterm birth, and congenital anomalies such as heart defects, hypospadias, and esophageal atresia.^9^ These patients should have a baseline evaluation for end-organ damage at their first prenatal visit, including the following tests: serum aspartate aminotransferase and alanine aminotransferase, serum creatinine, serum electrolytes, blood urea nitrogen, complete blood count, spot urine/creatinine or a 24-hour urine protein if the spot urine/creatinine is greater than 0.15, and an electrocardiogram.^1^

### Recommendations

Patients with chronic hypertension should undergo evaluation prior to pregnancy to allow for optimization of medical comorbidities and appropriate counseling regarding modifiable risk factors. Prepregnancy counseling should include a review of antihypertensive medications, with an emphasis on which medications should and should not be utilized in pregnancy (Table 1). Angiotensin-converting enzyme inhibitors and angiotensin receptor blockers should not be prescribed for patients desiring fertility because these treatments are teratogenic and may lead to fetal malformations and growth restriction.^10^

**Table 1:** Type, dosage, and timing of pharmacologic antihypertensive therapies during pregnancy. PO: by mouth; IV: intravenous; BP: blood pressure; BID: twice daily; q: every


MEDICATION	ROUTE	DOSAGE	MAXIMUM DOSAGE	TIMING OF THERAPY

Aspirin	PO	81 mg	N/A	Begin at 12-16 weeks of gestation and continue until delivery

Magnesium sulfate*	IV	Loading dose: 4-6 g bolus Maintenance dose: 2 g/hr if creatinine (Cr) ≤ 1.1; 1 g/hr if Cr > 1.1	N/A	When patient meets criteria for pre-eclampsia with severe features and continued 24 hours postpartum

Hydralazine	IV	5 or 10 mg, repeat q20 minutes if BP > 160/110. If still elevated, administer alternate medication	20 mg/day	For acute management until BP controlled

Labetalol	IV	Sequence: 20 mg, 40 mg, 80 mg q10 minutes if BP > 160/110	300 mg/day	For acute management until BP controlled

	PO	Begin with 200 mg BID and up-titrate as needed	2400 mg/day	Titrate to management of normotensive to mild range BP. Re-evaluate 1 week postpartum

Nifedipine immediate release (IR)	PO	10 mg	180 mg/day	For acute management of BP

Nifedipine extended release (XL)	PO	30 mg q daily and up-titrate as needed to 60 mg BID	120 mg/day	Titrate to management of normotensive to mild range BP. Re-evaluate 1 week postpartum


* For seizure prophylaxis

When patients with chronic hypertension do present in pregnancy, the baseline laboratory values should include the following: serum aspartate aminotransferase and alanine aminotransferase, serum creatinine, serum electrolytes (particularly potassium), blood urea nitrogen, complete blood count, spot urine/creatinine or 24-hour urine protein if the spot urine/creatinine shows abnormalities, and an electrocardiogram. A spot urine-to-protein creatinine ratio of less than 0.15 adequately equates to less than 300 mg of proteinuria on a 24-hour collection, and therefore there is no initial need for further evaluation. When women have baseline proteinuria and renal dysfunction is clinically suspected, it is associated with an increased risk for adverse pregnancy outcomes including an increased risk of superimposed preeclampsia (79% vs 49%), preterm delivery (48% vs 26% when less than 37 weeks gestation), delivering small-for-gestational-age infants, less than the 10th percentile (41% vs 22%), and neonatal admission to an intensive care unit.^1,11^

Women with poorly controlled hypertension for more than 4 years or who are older than 30 years of age are more likely to have cardiomegaly, ischemic heart disease, and cardiac hypertrophic changes—all problems that must be addressed to avoid further risk in the prenatal, childbirth, and postpartum periods.^1^ An electrocardiogram is a reasonable first-line exam; those with additional risk factors or an abnormal electrocardiogram should be further evaluated by a cardiologist and undergo an echocardiogram.

Approximately 10% of hypertension in adult patients is due to secondary hypertension as a result of an underlying medical etiology.^5^ In patients diagnosed with chronic hypertension at a young age, with resistance to treatment, or with a strong family history of kidney disease, an evaluation for secondary hypertension should be performed in conjunction with a Maternal Fetal Medicine specialist and other indicated medical specialists.^1^

Common oral antihypertensive medications that have been deemed as safe in pregnancy include labetalol, nifedipine, and methyldopa. Hydrochlorothiazide and other diuretics are considered second-line treatment. When pregnant patients present with acute-onset severe hypertension—with systolic blood pressures > 160 mm Hg and/or diastolic blood pressures > 110 mm Hg that persist for > 15 minutes—intravenous labetalol, hydralazine, or immediate-release nifedipine should be utilized. Drug selection may be individualized based on the contraindications and onset of action.^1^ The use of intravenous antihypertensives is critical in pregnancy because hypertensive encephalopathy or cerebrovascular accidents can occur without proper intervention.^12^ In the event of a true hypertensive emergency, with a blood pressure at or above 240/140 mm Hg, patients may develop significant end-organ damage and should be managed in an intensive care unit with a critical care physician, obstetrician, and maternal fetal medicine specialist to manage their care. Most institutions have established protocols and guidelines for the management of acute-onset severe hypertension in pregnancy. During the management of acute hypertension in pregnancy, it is critical to not over-aggressively medicate because it can result in maternal hypoperfusion and subsequent fetal heart rate abnormalities and hypoxia to the infant.^1,13^

While prior guidelines suggested initiation of antihypertensive medications with blood pressures > 160/110 mm Hg, a recently published study showed evidence to support the initiation of medications at a blood pressure of 140/90 mm Hg. The Chronic Hypertension and Pregnancy (CHAP) study published in the *New England Journal of Medicine* in April 2022 was a multicenter randomized trial that enrolled 2,408 patients with mild chronic hypertension (blood pressures < 160/110 mm Hg) and a singleton gestation. In this study, the intervention arm received oral antihypertensive medications beginning at a blood pressure of 140/90 mm Hg, and the control group did not receive any medications unless their blood pressure was > 160/105 mm Hg. The results of this study showed a reduction in the risk of primary outcomes including preeclampsia with severe features, medically indicated preterm birth less than 35 weeks gestation, placental abruption, or fetal or neonatal death. The incidence of a primary outcome in the treatment group was 37% versus 30.2% in the control group, for an adjusted risk ratio for 0.82. Given the above findings, the American College of Obstetricians and Gynecologists (ACOG) recommends using 140/90 mm Hg as the threshold for initiating antihypertensive therapy in pregnant patients with chronic hypertension.^14^ ACOG also recommends that women with an elevated risk of preeclampsia, including patients with chronic hypertension, should begin daily treatment with low-dose aspirin (81 mg) beginning at 12 weeks gestation and continuing until delivery to aid in the prevention of preeclampsia. Treatment beginning after 28 weeks of gestation is unlikely to provide maternal benefit.^15^

Antenatal fetal surveillance beginning at 32 weeks of gestation is recommended for pregnancies complicated by chronic hypertension, as are serial growth ultrasounds during the third trimester. Given the high-risk condition of these patients, continuous fetal monitoring should be performed throughout labor. This patient population has also been noted to have a longer first stage of labor, especially in the nulliparous population.^16^ ACOG recommends that pregnant patients with chronic hypertension deliver as follows: at 38.0 to 39.6 weeks of gestation for patients with controlled chronic hypertension and not requiring medications; at 37.0 to 39.6 weeks of gestation for controlled chronic hypertension requiring medications; and 36.0 to 37.6 weeks of gestation for patients whose chronic hypertension is not well controlled on medication.^3^ For women who develop superimposed preeclampsia with severe features and have abnormal laboratory values or symptomatology (described below), the recommendation is delivery by 34.0 weeks gestation.^1^

Disparities in hypertensive disorders play a significant role in the outcomes of several racial-ethnic groups. In an evaluation of historical trends over the past 40 years, the rate of maternal mortality related to hypertension was almost four-times higher in Black women than in White women, a figure that may reflect various forms of systemic racism in health care, such as poor access to care and social inequities.^17^

In October 2023, *The Green Journal*, one of the preeminent journals of obstetrics and gynecology research in the United States, published a special issue on the intersection of racism and reproductive health. One of the published reports, a population-based study evaluating 8 million pregnancies, found that when stratifying by ethnic group, 5.1% of Black patients and 2.9% Native Hawaiian or other Pacific Islander patients had chronic hypertension—and this contributed to a higher proportion of adverse outcomes including severe obstetric morbidity, acute renal failure, and stillbirth in these patients. The population attributable risk of a cerebrovascular accident was also highest in Black patients. This study further illustrates the impact of racial-ethnic disparities in patients with chronic hypertension in pregnancy.^18^

## Gestational Hypertension and Preeclampsia

### Background and Definitions

Hypertensive disorders of pregnancy, including gestational hypertension, preeclampsia without severe features, preeclampsia with severe features, eclampsia, and hemolysis, elevated liver enzymes, and low platelet count (HELLP) syndrome, affect 2% to 8% of pregnancies worldwide and contribute to approximately 16% of maternal mortality.^19^ Rates of all preeclampsia rose from 3.4% in 1980 to 3.8% in 2010, which is a result of an increase in severe preeclampsia from 0.3% in 1980 to 1.4% in 2010.^20^

Gestational hypertension is defined by systolic blood pressures of 140 mm Hg or greater or diastolic blood pressures of 90 mm Hg or greater on occasions greater than 4 hours apart after 20 weeks of gestation without proteinuria in patients with previously normotensive blood pressures. A diagnosis of preeclampsia without severe features is made by the presence of the above criteria, along with the presence of proteinuria, as defined by one of the following: 300 mg or more of protein in a 24 hour urine collection, a protein/creatinine ratio of 0.3 mg/dL or more, or a dipstick reading of 2+.^21^

A diagnosis of preeclampsia with severe features can be made in patients with systolic blood pressures of 160 mm Hg or greater or diastolic blood pressures of 110 mm Hg or greater on occasions greater than 4 hours apart after 20 weeks of gestation with previously normotensive blood pressures and proteinuria *or* one of the following criteria: thrombocytopenia (platelet count less than 100k/uL); twice the upper level of normal for aspartate aminotransferase (AST) and alanine aminotransferase (ALT) that cannot be attributed to another diagnosis; serum creatinine greater than 1.1 mg/d or double a patient’s baseline; pulmonary edema; and/or new-onset headache unresponsive to pain medication that cannot be attributed to another diagnosis. A diagnosis of preeclampsia with severe features can also be made with persistently elevated systolic blood pressures of 160 mm Hg or greater or diastolic blood pressures of 110 mm Hg at least 15 minutes apart, along with severe persistent right upper quadrant pain or epigastric pain unresponsive to pain medication and visual disturbances such as scotoma.^21^

Risk factors for preeclampsia include nulliparity, multifetal gestations, a history of preeclampsia in prior pregnancy, chronic hypertension, gestational and pregestational diabetes, thrombophilia, systemic lupus erythematosus, prepregnancy body mass index greater than 30 kg/m^2^, antiphospholipid antibody syndrome, advanced maternal age (35 years or older), kidney disease, assisted reproductive technology, and obstructive sleep apnea.^21^

HELLP syndrome is diagnosed by the following criteria: lactate dehydrogenase (LDH) elevated to 600 IU/L or more, AST and ALT elevated more than twice the upper limit of normal, and platelets count less than 100k/uL. Eclampsia is defined as new-onset tonic-clonic, focal, or multifocal seizures without other explanative diagnoses, medications, or drugs in circulation. It is a significant cause of maternal mortality and morbidity.^21^ Examples of morbidity include aspiration pneumonia, neurological damage like short-term or long-term memory loss, or cognitive dysfunction.^22^

Gestational hypertension and preeclampsia are proposed to be on a spectrum of disease rather than separate disease processes, with up to 50% of patients initially diagnosed with gestational hypertension developing criteria that meet the diagnosis of preeclampsia, especially when diagnosed earlier in pregnancy (< 32 weeks gestation).^21^ However, despite nomenclature, very few patients diagnosed with preeclampsia go on to develop eclampsia, with or without magnesium sulfate prophylaxis, and one study even showed that 38% of patients diagnosed with eclampsia had no previous hypertensive blood pressures or proteinuria.^23^

Possible hypotheses for the pathophysiology of preeclampsia spectrum diagnoses include chronic uteroplacental ischemia, immune maladaptation, very-low-density lipoprotein toxicity, genomic imprinting, increased trophoblast apoptosis or necrosis, an exaggerated maternal inflammatory response to deported trophoblasts, and, most recently, imbalances of angiogenic factors.^24,25,26,27,28,29^ Patients with preeclampsia lack the typical physiologic change of hypervolemia in pregnancy, resulting in hemoconcentration of circulating blood. They also have vasospastic vessels due to an imbalance of circulating vasoactive and vasoconstrictive agents. Therefore, the systemic vasospasm of blood vessels can lead to a decrease in volume in the intravascular space, leading to renal system retention of water and sodium in preeclamptic patients with a transient oliguria in labor and the first 24 hours postpartum. This decrease in intravascular volume can result in a hemoconcentration and may lead to an inaccurate assessment of a patient’s hematocrit. For example, in the case of HELLP syndrome, the suspected presence of hemolysis should be confirmed with an elevated lactate dehydrogenase level in the setting of anemia.^21^

Many of the fetal consequences of preeclampsia are secondary to decreased uteroplacental blood due to a lack of spiral artery transformation or placental bed vascular formation. Such consequences include fetal growth restriction, oligohydramnios, placental abruption, and nonreassuring fetal status on antenatal testing and subsequent increased risk of spontaneous or indicated preterm delivery. Long-term complications of fetal growth restriction include the development of cognitive delay in childhood and chronic diseases in adulthood, including obesity, type two diabetes, stroke, and coronary artery disease.^21,30^

### Recommendations

Low-dose aspirin is the only evidence-based prevention strategy for hypertensive disorders of pregnancy.^15^ Ideally, it should be initiated before 16 weeks of gestation, but anywhere from 12 to 28 weeks, for a more significant reduction in severe preeclampsia and fetal growth restriction. If a pregnant patient has at least one high-risk factor (previous pregnancy with preeclampsia, multifetal gestation, renal disease, autoimmune disease, type 1 or 2 diabetes mellitus, and chronic hypertension) or more than one moderate risk factor (first pregnancy, maternal age of 35 years or older, a body mass index > 30 kg/m^2^, family history of preeclampsia, sociodemographic characteristics, and personal history factors), they should receive 81 mg of aspirin daily until delivery for hypertensive disease reduction.^15^

ACOG recommends delivery for pregnant patients with hypertensive disease, as follows: at 37 weeks of gestation or at the time of diagnosis if later for patients with gestational hypertension or preeclampsia without severe features; at 34 weeks of gestation or at the time of diagnosis if later for patients with preeclampsia with severe features if mother and fetus are stable. If the patient has preeclampsia with severe features or HELLP and/or additional conditions or features that make them unstable, the recommendation is delivery after stabilization whether or not the fetus is viable.^21^

Outpatient antenatal testing is recommended for patients diagnosed with gestational hypertension or preeclampsia without severe features starting at 32 weeks gestation. Antenatal surveillance consists of serial fetal growth ultrasounds every 3 to 4 weeks and weekly amniotic fluid and antenatal testing (typically a biophysical profile). Similarly, preeclampsia labs, particularly platelet count, serum creatinine, and liver enzyme levels, should be repeated on a weekly basis, or sooner if indicated.^21^

Magnesium sulfate is recommended by ACOG for seizure prophylaxis in patients diagnosed with preeclampsia with severe features or eclampsia unless a patient has a condition that is contraindicatory, such as myasthenia gravis, hypocalcemia, moderate-to-severe renal failure, cardiac ischemia, heart block, or myocarditis. Intravenous administration is generally preferred to intramuscular administration secondary to the lower rate of adverse effects. Urine output, respiration status, and deep tendon reflexes should all be routinely monitored during magnesium sulfate administration. Medication should be continued 24 hours after delivery in both vaginal and Cesarean deliveries.^21^

The same medications and considerations mentioned previously for chronic hypertension and superimposed preeclampsia should be used for the management of severe blood pressures in hypertensive disorders of pregnancy. For preeclamptic patients, it is not recommended to try to correct hypovolemia with intravenous fluids because frequent capillary leak and increased colloid pressure can lead to increased third spacing of fluids and increase the risk of pulmonary edema.^21^

Despite the lab abnormalities associated with the diagnoses, neuraxial anesthesia is still the preferred method of anesthesia for women with preeclampsia with severe features and eclampsia for labor and delivery. Episodes of hypotension are treatable with medication. and the only true contraindication is platelet counts < 70 × 10^9^/L due to the risk of epidural hematoma.^31^

ACOG recommends the use of nonsteroidal anti-inflammatory drugs (NSAIDs) in patients with hypertensive diagnoses in pregnancy during the immediate postpartum period despite the medications’ mode of action, which could potentially exacerbate disease processes. For example, NSAIDs lead to a decrease in prostaglandins and a subsequent lack of vasodilation and an increase in sodium retention, which could theoretically increase a patient’s blood pressure. Studies have shown that use of these medications for pain control not only decreases the use of opioids but also does not lead to an increased duration of severe-range blood pressures, differences in blood pressure, antihypertensive requirements, or other adverse events.^32^

Patients with a history of preeclampsia have double the risk of cardiovascular disease outside of pregnancy, including myocardial infarction, congestive heart failure, cerebrovascular events, and peripheral arterial disease, and five times the risk of chronic hypertension.^33^ Patients with more severe cases of preeclampsia or eclampsia have increasingly higher rates of cardiovascular disease and hypertension than their pregnant counterparts without hypertensive diagnoses in pregnancy.^33^ Endothelial dysfunction in preeclampsia persists in patients for years after delivery and can provide a potential explanation for this increased risk, although prepregnancy cardiovascular risk factors explain approximately half of the increased risk.^34^

One theory used to explain the increased risk of cardiovascular disease in women with preeclampsia postulates that pregnancy is a “stress test” and women who develop preeclampsia likely will develop cardiovascular disease in the future.^35^ This theory is derived from the similar risk factors of preeclampsia and cardiovascular disease.

A retrospective cohort study entitled Cardiovascular Health After Maternal Placental Syndrome (CHAMPS) was completed in Canada and evaluated a total of 1.03 million women who experience a maternal placental syndrome such as gestational hypertension, placental abruption, and placental infarction. The outcomes showed an increased risk of coronary artery disease, peripheral vascular disease, and cerebrovascular disease in these women. A more pronounced risk of cardiovascular disease was noted in women with poor fetal growth, and even worse with fetal death, signifying an elevated risk of cardiovascular disease in those with severe placental disease.^36^

In stratifying cardiovascular disease risk, there is a linear relationship between a patient’s prepregnancy body mass index and hypertriglyceridemia and the risk of preeclampsia.^37,38^ These markers of metabolic dysfunction may not only proceed a pregnancy affected by maternal placental syndrome but may also appear postpartum. Therefore, weight management and subsequent management of metabolic syndromes may be critical to mitigating a woman’s risk of developing both maternal placental disease and cardiovascular disease. Postpartum evaluation of these patients within 6 months should include a blood pressure measurement, weight, abdominal circumference, and potentially a lipid panel and measurement of serum glucose as an opportunity to identify and evaluate an at-risk patient as early as possible.^39,40^

## Key Points

- The diagnosis of chronic hypertension, gestational hypertension, and preeclampsia is vital in pregnancy due to the potential morbidity and mortality risks to maternal and fetal outcomes.
- Typical physiological cardiovascular changes associated with pregnancy can impact the presentation and sequelae of chronic hypertension.
- The clinical considerations, treatment, and management of these diagnoses include the judicious use of aspirin for preeclampsia prevention, baseline and serial laboratory testing, antenatal fetal monitoring, and initiation of antihypertensives with blood pressures of 140/90 mm Hg.
